# Sympathy for the Erring

**Published:** 1857-07

**Authors:** 


### SYMPATHY FOR THE ERRING.

Of how much of our indignation against even a deliberate wrong would we be disarmed, if we could but know for ourselves a tithe of all the sorrow, and trouble, and disappointment the poor erring heart had passed through! What efforts were made in youth to stand up against the pressure of the world, and how, when fallen, from miscalculation, or an over confiding nature or want of tact, it bravely rose up and tried again; and when hard necessity came and drove it to the wall, how it looked around for help, and waited, still striving to stand upright, and fell while striving, and even when fallen, how it yearned for one more chance to rise and be a man, how loth at at last to give up all for lost!—could we but see a thousandth part of these struggles, as they rend our brother's bosom, and almost break his heart, how should it disarm us of our vindictiveness, and incline us even to run to him, and raise him up, and stand by him, and with godlike forgiveness, bid him, in tones of encouragement, "Try, try again!"

